# First study on molecular detection of hemopathogens in tabanid flies (Diptera: Tabanidae) and cattle in Southern Thailand

**DOI:** 10.14202/vetworld.2022.2089-2094

**Published:** 2022-08-28

**Authors:** Narin Sontigun, Worakan Boonhoh, Yotsapat Phetcharat, Tuempong Wongtawan

**Affiliations:** 1 Akkhraratchakumari Veterinary College, Walailak University, Nakhon Si Thammarat 80160, Thailand; 2 One Health Research Center, Walailak University, Nakhon Si Thammarat 80160, Thailand; 3 Centre of Excellence Research for Melioidosis and Other Microorganisms, Walailak University, Nakhon Si Thammarat 80160, Thailand

**Keywords:** cattle, hemopathogen, tabanids, Thailand, vector

## Abstract

**Background and Aim:**

Female tabanids play a key role in disease transmission as mechanical vectors for various hemopathogens, but only a limited number of studies have been conducted on them. This study aimed to investigate the occurrence of hemopathogens in tabanid flies compared to those found in nearby cattle hosts.

**Materials and Methods:**

Tabanids were collected using a Nzi trap for three consecutive days per month during the dry season (February–May 2021). Furthermore, blood samples were collected from 20 beef cattle (*Bos taurus*) raised in the same area where the flies were captured. Conventional polymerase chain reaction (PCR) was used to detect hemopathogenic DNA in flies and beef cattle.

**Results:**

In total, 279 female tabanids belonging to five species were collected: *Tabanus megalops*, *Tabanus rubidus*, *Tabanus mesogaeus*, *Chrysops dispar*, and *Chrysops fuscomarginalis*. Notably, *T. megalops* was the most abundant species, accounting for 89.2% of the flies collected (n = 249). PCR technique revealed that 76.6% of *T. megalops* carried at least one pathogen (*Anaplasma, Ehrlichia, Babesia, or Theileria*). In addition, all beef cattle had multiple hemopathogenic infections (*Anaplasma marginale*, *Ehrlichia* spp., *Babesia bigemina*, *Babesia bovis*, and *Theileria* spp.).

**Conclusion:**

Although *T. megalops* could carry many hemopathogens, it might not be an important vector due to the limited number of flies and parasitic load. Furthermore, *T. megalops* could be utilized to monitor the presence of hemopathogens in the study area, but not the disease occurrence in the individual host species. Knowing the presence of hemopathogens in flies could help manage the disease in this area.

## Introduction

The family Tabanidae (Diptera) is a group of blood-sucking flies that can harm animal and human health [1–3]. Female tabanids of most species can bite and consume animal blood, causing discomfort, pain, skin lesions, and blood loss in animals, resulting in a reduction in livestock productivity [4]. In addition, they serve as mechanical vectors for a variety of pathogens that affect human and animal health, including viruses (e.g., equine infectious anemia virus, lumpy skin disease, and bovine leukosis virus), bacteria (e.g., *Anaplasma marginale*, *Anaplasma phagocytophilum*, *Brucella* spp., and *Listeria monocytogenes*), and protozoa (e.g., *Besnoitia besnoiti*, *Babesia bigemina*, *Trypanosoma* spp., *Theileria* spp., and *Leishmania donovani*) [5–12]. Tabanid flies are also biological vectors for *Trypanosoma* (*Megatrypanum*) *theileri* [5, 13, 14]. Furthermore, some species act as intermediate hosts for filarial nematodes, such as *Dirofilaria repens*, *Dirofilaria roemeri*, *Elaeophora schneideri*, as well as *Loa loa* that causes loiasis (African eye worm) in humans [5, 15]. Hemopathogens in the genera *Anaplasma*, *Ehrlichia*, *Babesia*, *Theileria*, and *Trypanosoma* are the most economically significant pathogens of livestock worldwide, particularly in tropical and subtropical areas [16–19]. Most of them, except for *Trypanosoma* spp., are biologically transmitted by ticks, but they are also mechanically transmitted by flies such as stomoxys and tabanids [5, 20].

In Thailand, hemopathogens have been usually investigated in the host animals (e.g., cattle, buffalos, and goats) and ticks, the main biological vectors [16, 21–23]. To date, approximately 128 species of tabanids have been identified in Thailand, including the genera *Tabanus* (86 species), *Haematopota* (36 species), and *Chrysops* (6 species) [24–26], among which the three most abundant species were *Tabanus striatus*, *Tabanus megalops*, and *Tabanus rubidus*, respectively [27]. Although tabanids may play an important role as disease vectors for animals and humans, they have received little attention compared to stomoxys flies [28–30]. Only a few studies on hemopathogens in tabanids have been documented in Thailand [8, 9, 31], and the data are still limited. Furthermore, no evidence is available regarding the presence of hemopathogens in tabanids in Southern Thailand.

The present study aimed to investigate the occurrence of hemopathogens in tabanid flies using conventional polymerase chain reaction (PCR) and to validate whether the presence of hemopathogens in the biting flies could be used as a monitoring marker. The findings of this study may help determine the role of tabanids in disease transmission and the risk of infection to animals in these areas, leading to the design of effective vector control and improvement of animal disease management.

## Materials and Methods

### Ethical approval

This study was approved by the Institutional Animal Care and Use Committee of Walailak University (Project number WU-AICUC-63-015).

### Study period and location

The samples were collected from February to May 2021 in Nakhon Si Thammarat province, Southern Thailand. 

### Tabanid fly collection and species identification

**Figure-1:**
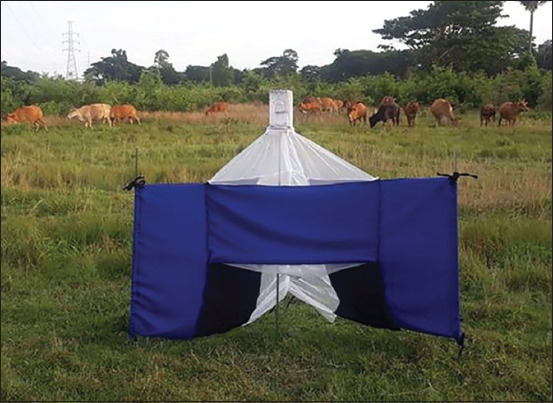
Nzi trap used for fly collections.

Adult tabanids were captured during the dry season (February–May 2021) using a Nzi trap [32] (Figure-1) placed in a beef cattle farm (N 8°38′40.5672″, E 99°54′07.3296″) located in Thasara district, Nakhon Si Thammarat province. A trap was placed from 6.00 a.m. to 6.00 p.m. for three consecutive days each month. Flies were collected twice a day and transported to the Parasitology Laboratory at Akkhraratchakumari Veterinary College, Walailak University. In the laboratory, samples were frozen for 15 min at −40°C and then morphologically identified to the species level using a stereomicroscope (Olympus, Tokyo, Japan) based on the taxonomic keys of Burton [25] and Burger and Chainey [33]. The identified specimens were kept individually in a 1.5 mL microcentrifuge tube containing 95% ethanol and stored at −40°C for further analysis.

### Blood collection from the beef cattle

Blood samples were collected from the jugular vein of 20 beef cattle (*Bos taurus*) raised in the same area where the flies were captured. Blood samples (3 mL each) were collected in 3-mL vacuum tubes containing ethylenediaminetetraacetic acid K3 and transferred to the laboratory in a cooler containing ice packs.

### DNA extraction

The ethanol-preserved tabanid specimens were air-dried, and genomic DNA was extracted from a combination of three body parts (i.e., the head with mouthparts, thorax, and abdomen) of individual specimens using the E.Z.N.A.^®^ Tissue DNA Kit (Omega Bio-Tek, Norcross, Georgia, USA), according to the manufacturer’s instructions. For blood samples, genomic DNA was extracted from 200 mL of blood using the E.Z.N.A.^®^ Blood DNA Kit (Omega Bio-Tek). The concentration of extracted DNA was measured using Nano-Drop™ One C Microvolume UV-Vis spectrophotometer (Thermo Fisher Scientific, Massachusetts, USA) and then stored at −20°C until further analysis.

### Hemopathogen detection

Conventional PCR was used to detect hemopathogenic DNA in flies and beef cattle. Individual specimens were first screened using genus-specific primers to detect *Anaplasma* spp., *Ehrlichia* spp., *Babesia* spp., *Theileria* spp., and *Trypanosoma* spp. Subsequently, PCR-positive samples were analyzed using species-specific primers to detect the common hemopathogen species (*A. marginale*, *B. bigemina*, *Babesia bovis*, and *Theileria orientalis*) found in cattle in Thailand.

The PCR reaction contained 6.25 µL DreamTaq Green Master Mix (2×) (Thermo Scientific, Vilnius, Lithuania), 1–2 µL DNA template (100–200 ng/µL), primer at various concentrations (Table-1) [34–41] and nuclease-free water to a final volume of 12.5 µL. PCR was performed using the Mastercycler Pro S machine (Eppendorf AG, Hamburg, Germany). Details of the primers and primer concentration are listed in Table-1. The PCR cycle comprised an initial denaturation step at 95°C for 3 min followed by 35 cycles of denaturation at 95°C for 30 s. Annealing temperatures were 55°C for *Babesia* spp.; 57°C for *Ehrlichia* spp.; 58°C for *Anaplasma* spp., *Theileria* spp., *Trypanosoma* spp., *A. marginale*, *B. bigemina*, and *T. orientalis*; and 62°C for *B. bovis* for 30 s. An initial extension step was performed at 72°C for 1 min (except for *Babesia* spp., which used 30 s), and a final extension was done at 72°C for 5 min. For each assay, the genomic DNA samples from known blood pathogens were used as positive controls, while the nuclease-free water was used as a negative control. The PCR products were electrophoretically separated on 2% agarose gel in 1× TAE buffer, stained with SERVA DNA Stain G (SERVA, Heidelberg, Germany), and visualized under UV light using the ChemiDoc™ Imaging System (Bio-Rad, USA).

**Table-1:** Details of primers and primer concentrations for hemopathogen detection.

Pathogen	Gene	Primer name	Sequences (5’to 3’)	Product size (bp)	Primer concentration (mM)	Reference
*Anaplasma* spp.	16S rRNA	HGE162F HGE162R	TAGTAGTATGGGATAGCCACTAGAA GTGTGGCTGATCATCCT	166	0.4	[34]
*Babesia* spp.	lsu5-lsu4	B-lsu-F B-lsu-R2	ACCTGTCAARTTCCTTCACTAAMTT TCTTAACCCAACTCACGTACCA	180	0.4	[35]
*Ehrlichia* spp.	16S rRNA	EHR163F EHR163R	GCCTACGTTAGATTAGCTAGTTG CTGGATCAGGCTTTCGC	163	1	[34]
*Theileria* spp.	18S rRNA	THEIFP THEIRP	TAGTGACAAGAAATAACAATACGGGGCT CAGCAGAAATTCAACTACGAGCTTTTTAACT	180	0.4	[36]
*Trypanosoma* spp.	ITS1	ITS1 CF ITS1 BR	CCGGAAGTTCACCGATATTG TGCTGCGTTCTTCAACGAA	250–710	1	[37]
*Anaplasma marginale*	MSP4	AmargMSP4Fw AmargMSP4Rev	CTGAAGGGGGAGTAATGGG GGTAATAGCTGCCAGAGATTCC	344	0.4	[38]
*Babesia bovis*	VESA–1a	bovar2AF bovar2AR	CAAGCATACAACCAGGTGG ACCCCAGGCACATCCAGCTA	166	0.4	[39]
*Babesia bigemina*	18S rRNA	Bg_18S32F Bg_18S150R	GGAATGATGGTGATGTACAACCTCA CGCGAGGCTGAAATACAACTACG	147	0.4	[40]
*Theileria orientalis*	MPSP	MPSP-F MPSP-R	CTTTGCCTAGGATACTTCCT ACGGCAAGTGGTGAGAACT	776	0.4	[41]

## Results

### Diversity of tabanids

Among the 279 female tabanids collected, the most abundant species was *T. megalops* (89.2%; n = 249). Others were *T. rubidus* (8.2%; n = 23), *Tabanus mesogaeus* (1.1%; n = 3), *Chrysops dispar* (1.1%; n = 3), and *Chrysops fuscomarginalis* (0.4%; n = 1).

### Hemopathogen detection in *T. megalops*

Among 128 samples analyzed by PCR using genus-specific primers, hemopathogens were detected in 76.6% (n = 98). Of the 98 positive samples, 39 flies (30.5%) were positive for a single pathogen, 49 flies (38.3%) were positive for two pathogens, nine flies (7%) were positive for three pathogens, and one fly (0.8%) was positive for four pathogens (Table-2). *Trypanosoma* DNA was undetectable. The co-existence of *Ehrlichia* spp. and *Theileria* spp. in a single fly was predominant (34.4%, n = 44), followed by that of a single pathogen, *Theileria* spp. (28.1%, n = 36). Subsequently, the positive samples were reanalyzed with species-specific primers to determine the presence of *A. marginale*, *B. bigemina*, *B. bovis*, and *T. orientalis*; however, none was detected.

**Table-2:** Hemopathogen detection in *Tabanus megalops* (n=128) collected from a beef cattle farm.

Hemopathogen	No. of positive samples	Prevalence
Single pathogen		
*Anaplasma* spp.	2	1.6%
*Ehrlichia* spp.	1	0.8%
*Theileria* spp.	36	28.1%
Two pathogens		
*Anaplasma* spp. and *Ehrlichia* spp.	1	0.8%
*Anaplasma* spp. and *Theileria* spp.	2	1.6%
*Ehrlichia* spp. and *Theileria* spp.	44	34.4%
*Babesia* spp. and *Theileria* spp.	2	1.6%
Three pathogens		
*Anaplasma* spp., *Ehrlichia* spp., and *Theileria* spp.	5	3.9%
*Ehrlichia* spp., *Babesia* spp., and *Theileria* spp.	4	3.1%
Four pathogens		
*Anaplasma* spp., *Ehrlichia* spp., *Babesia* spp., and *Theileria* spp.	1	0.8%
Total	98	76.6%

### Hemopathogen detection in beef cattle

All (n = 20) cattle were found to be infected with hemopathogens such as *Anaplasma*, *Ehrlichia*, *Babesia*, and *Theileri*a, but not *Trypanosoma* (Table-3). Among species-specific pathogens, *A. marginale*, *B. bigemina*, and *B. bovis* were found. Furthermore, multiple infections were detected in all cattle; nine samples (45%) were infected with four pathogens (*A. marginale*, *Ehrlichia* spp., *B. bigemina* or *B. bovis*, and *Theileria* spp.), and 11 samples (55%) were infected with five pathogens (*A. marginale*, *Ehrlichia* spp., *B. bigemina*, *B. bovis*, and *Theileria* spp.).

**Table-3:** Hemopathogen detection in beef cattle (n=20) collected from a beef cattle farm.

Hemopathogen	No. of positive samples	Prevalence
Four pathogens		
*A. marginale, Ehrlichia* spp., *B. bigemina, Theileria* spp.	4	20%
*A. marginale, Ehrlichia* spp., *B. bovis, Theileria* spp.	5	25%
Five pathogens		
*A. marginale, Ehrlichia* spp., *B. bigemina*, *B. bovis*, *Theileria* spp.	11	55%
Total	20	100%

*A. marginale=Anaplasma marginale, B. bigemina=Babesia bigemina, B. bovis=Babesia bovis*

### The occurrence of each pathogen

The occurrence of each pathogen is shown in Table-4. For *T. megalops* (n = 128), the highest occurrence of hemopathogens was that of *Theileria* spp. at 73.4% (n = 94), followed by 43.8% of *Ehrlichia* spp., 8.6% of *Anaplasma* spp., and 5.5% of *Babesia* spp.

**Table-4:** Frequencies of detected hemopathogens in *Tabanus megalops* (n=128) and beef cattle (n=20) collected from a beef cattle farm.

Organism	Hemopathogen	No. of positive samples	Percentage
*Tabanus megalops*	*Anaplasma* spp.	11	8.6
	*Babesia* spp.	7	5.5
	*Ehrlichia* spp.	56	43.8
	*Theileria* spp.	94	73.4
Beef cattle	*Anaplasma* spp.	20	100
	*Babesia* spp.	20	100
	*Ehrlichia* spp.	20	100
	*Theileria* spp.	20	100
	*Anaplasma marginale*	20	100
	*Babesia bovis*	15	75
	*Babesia bigemina*	15	75

In cattle (n = 20), the occurrence of hemopathogen infection was 100% for the genera *Anaplasma* spp., *Theileria* spp., *Ehrlichia* spp., and *Babesia* spp. In terms of species-specific pathogenic occurrence, *A. marginale* was found at 100%, whereas *B. bovis* and *B. bigemina* were found at 75% (n = 15).

## Discussion

The present study revealed that *T. megalops* was the most abundant tabanid in the study area. Tabanids were found to carry many hemopathogens (*Anaplasma*, *Ehrlichia*, *Babesia*, and *Theileria*). In addition, all hemopathogens detected in the flies were also found in cattle, suggesting that detection of hemopathogens in flies could be used as a convenient tool for monitoring diseases in the area. However, the occurrence of hemopathogens in cattle was higher than that in biting files, implying that the flies might bite other animals around the area. Therefore, we suggest using flies to determine the presence of hemopathogens in a given area but not the occurrence of diseases.

This is the first study on hemopathogens in tabanids in Southern Thailand, revealing that *T. megalops* can harbor many hemopathogen genera. In contrast, a previous study reported the presence of only one genus, *Theileria* [8]. Other tabanid species in Thailand, including *T. rubidus* and *T. striatus*, are capable of carrying *A. marginale* [9]. Different groups of biting flies such as Stomoxyinae (i.e., *Stomoxys calcitrans*, *Stomoxys indicus*, *Stomoxys pullus*, *Stomoxys sitiens*, *Haematobosca sanguinolenta*, and *Haematostoma austeni*) can carry several hemopathogens such as *Theileria* spp., *Babesia canis vogeli* [31], and *A. marginale* [9].

The present study revealed that most *T. megalops* carried *Anaplasma* spp., *Ehrlichia* spp., *Babesia* spp., and *Theileria* spp.; these hemopathogens were also found in the beef cattle nearby. The occurrence of hemopathogens in cattle (100%) was higher than that reported in a previous study (42.5%) conducted in eight provinces (Songkhla, Pattalung, Satun, Nakhon Si Thammarat, Pattani, Yala, Trang, and Narathiwat) in Southern Thailand, wherein *Theileria* spp. (37.53%) was the most commonly found [42]. The considerable difference between the two studies in terms of infection occurrence could be explained by the detection method used; our study used PCR, whereas the other study used microscopy. For the detection of hemopathogens, PCR is more sensitive than microscopy [17, 43].

*Babesia* spp., *A. marginale*, *T. orientalis*, and *Trypanosoma evansi* were previously reported to affect the health and productivity of cattle in tropical and subtropical areas, including Thailand [16–19]. One report revealed multiple infections with *B. bigemina, B. bovis*, *A. marginale*, and *T. orientalis* [16], similar to our findings. However, we additionally found the hitherto unreported high occurrence of *Ehrlichia* species in both flies (46%) and cattle (100%) in Thailand. Only four *Ehrlichia* species, namely, *Ehrlichia canis*, *Ehrlichia equi, Ehrlichia chaffeensi*, and *Ehrlichia risticii* have been reported in dogs in Thailand [44, 45]. Ehrlichiosis can have a negative impact on animal health and economics [46], and since this study used only genus-specific primers to identify *Ehrlichia*, additional research such as DNA sequencing or species-specific PCR should be conducted to gain comprehensive information that can help manage this disease in Thailand.

In the present study, *A. marginale*, *B. bigemina*, and *B. bovis* were not detected in *T. megalops* but found in the cattle. This discrepancy could be explained by the fact that the amount of DNA extracted from the flies was less than that found in bovine blood, or that the flies might have been carrying other species, as genus-specific primers detected PCR-positive results.

As previously noted by Foil [47], the characteristics of tabanids that make them good mechanical vectors include their large mouthparts, long-distance mobility, and their frequently interrupted feeding. The mode of mechanical transmission can occur due to the contamination of mouthparts or regurgitation of previously ingested blood during a blood meal. Notably, the success of mechanical transmission of hemopathogens by tabanids is dependent on the titers of pathogens, blood meal size, vector density, host density, and the survival of pathogens on the mouthparts and inside the gut of vectors. In this study, we did not quantify the amount of each pathogen per insect, but the negative results of PCR for each parasite species suggest that the pathogen load might be extremely low. Moreover, the number of captured tabanids was also low compared to the nearby host animals (more than 200 animals). These collective results lead us to believe that the possibility of a mechanical vector for *T. megalops* may be low.

There are many tabanid species in Thailand, but very little is known about their feeding behavior, host range, host preferences, and disease carriers. Since tabanids feed on the blood of a wide variety of vertebrate hosts, particularly livestock such as cattle, buffaloes, horses, goats, and sheep [2, 3], knowledge of the host preferences of individual tabanid species might play an essential role in the disease epidemiology. It would be beneficial in developing strategies for controlling diseases and vectors. Therefore, future studies should focus on blood meal source identification and the investigation of hemopathogens in more study areas across the country. In addition, monthly investigations may be carried out to detect the presence of hemopathogens in tabanid flies as well as in the blood of cattle. This would increase the reliability of using tabanids as a marker for the circulation of hemopathogens in the study area.

## Conclusion

The present investigation reveals that while the tabanid fly, *T. megalops*, may be a carrier of various infections (caused by *Anaplasma* spp., *Ehrlichia* spp., *Babesia* spp., and *Theileria* spp.), its contribution to the circulation of hemopathogens in the area of study may be minimal. Consequently, *T. megalops* may be utilized as a marker for the presence of hemopathogens in the area, allowing for more effective vector control and disease management in animals. Furthermore, because *Ehrlichia* spp. in cattle has never been reported in Thailand, the emergence of this infection needs to be urgently investigated to control it.

## Authors’ Contributions

NS: Conceptualization, methodology, validation, formal analysis, investigation, resources, data curation, writing–original draft preparation, writing–review and editing, visualization. WB: Investigation. YP: Investigation, resources. TW: Conceptualization, methodology, writing–original draft preparation, writing–review and editing, supervision. All authors have read and approved the final manuscript.
